# Editorial: Fibrous Assemblies: From Synthesis and Nanostructure Characterization to Materials Development and Application

**DOI:** 10.3389/fbioe.2021.778094

**Published:** 2021-10-01

**Authors:** Cinzia Giannini, Ulf Olsson, Maria Grazia Raucci, Dimitrios I Zeugolis

**Affiliations:** ^1^ Institute of Crystallography, National Research Council (IC-CNR), Bari, Italy; ^2^ Physical Chemistry Department, Lund University, Lund, Sweden; ^3^ Institute of Polymers, Composite and Biomaterials, National Research Council (IPCB-CNR), Naples, Italy; ^4^ Regenerative, Modular & Developmental Engineering Laboratory (REMODEL), Charles Institute of Dermatology, Conway Institute of Biomolecular & Biomedical Research and School of Mechanical & Materials Engineering, University College Dublin, Dublin, Ireland

**Keywords:** fibers, SAXS, WAXS, amyloids, Type1 collagen, elastin, fibroin

Fibrous assemblies are ubiquitous in living organisms, providing mechanical stability, elasticity and conservation of shape from cells to tissue and the full organism. In the human body, microtubules and actin filaments shape cells through the cytoskeleton and support the long projected axons of neurons. Collagen is the most abundant extracellular matrix protein in mammals. This structural protein is an important component, responsible for the unique mechanical properties of skin, tendon, cornea, cartilage, and bone. Elastin is responsible for the elastic properties of connective tissue. Actin and myosin filaments are basic components of skeletal muscle fibres. These amazing tissue materials have for a very long time inspired scientists to develop various biomimetic materials and to utilise such materials in tissue engineering. Other than physiological function, fibrous assemblies in our body can also be pathogenic. For example, the formation of peptide amyloid fibrils in the brain is associated with a number of different neurodegenerative diseases, the two most common of which being Alzheimer’s disease and Parkinson’s disease. Understanding these natural fibrous assemblies, on a fundamental level, is thus important for the development of biocompatible and biomimetic materials for a diverse range of clinical indication and to combat neurodegenerative diseases.

In the research topic *Fibrous Assemblies: From Synthesis and Nanostructure Characterization to Materials Development and Application*, research advances and limitations of fibrous assemblies are discussed. In a way, this research topic goes very much back to Leonardo da Vinci’s work 600 years ago, and this is the reason for our choice of the figure which illustrates the topic. Leonardo was, fascinated by bones and muscles, and interested in analysing the human body in mechanical terms. In the winter of 1,510–1,511, he participated in a number of autopsies at the University of Pavia, from which he skilfully produced many beautiful and fundamental tutorial drawings that also tell us how close Leonardo actually came to today’s modern medical thinking. Contributions to the topic cover different aspects of fibrous assemblies, ranging from their characterisation to contribution to reparative medicine and disease manifestation and progression. Fichman and Schneider discuss ways to increase the mechanical rigidity of supramolecular peptide-based gel systems using Frémy’s salt (). Liu et al. assess *in vivo* fibrous collagen dura substitutes. Nitti et al. demonstrate the potential of a magnesium doped hydroxyapatite/collagen scaffold in bone tissue engineering . Balasco et al. present a comprehensive review of amyloid-like aggregation in disease and biomaterials. Ozsvar et al. and Rodriguez-Cabello et al. provide a comprehensive review in tropoelastin and elastin assembly and the use of elastin-based biomaterials in regenerative medicine. Salvatore et al. discuss the utilisation of collagen-based materials in tissue repair and regeneration. Rizzo et al. investigates the behaviour of degummed silk fibroin in the presence of lanthanide ions. In a more fundamental level, Narayanan et al. conduct multiscale structural analysis of peptide nanotubes using X-Ray scattering methods and investigate amyloid peptide nanotube assembly using SAXS/WAXS methods (Narayanan et al.). Evidently, fibrous assemblies are fundamental in tissue genesis, disease and repair. Understanding the mechanism involved in these processes will enable us to develop tissue-specific facsimiles with enhanced therapeutic capacity.

